# Use of Sitters for Patient Safety in a Veteran Population

**DOI:** 10.1093/geroni/igaa057.971

**Published:** 2020-12-16

**Authors:** Denise Kresevic, Christopher Burant

**Affiliations:** 1 Louis Stokes Cleveland VA Medical Center, Sagamore Hills, Ohio, United States; 2 Case Western Reserve University, Parma, Ohio, United States

## Abstract

Patient safety including falls risk, is a high priority and an increasing challenge for all health care facilities. Safety risk factors include both physical factors and psychological factors. One common strategy to increase safety has been the use of “sitters.”. Studies on functions and outcomes are conflicting some have reported no differences in falls, decreases in falls and restraints, and increases in falls. A total survey sample of 22 “sitters” and 56 registered nurses conducted at a large midwestern VA facility to assess perceptions of sitters. Groups were similar in ages (41-50 years) with sitters having slightly more experience (11-20 years) versus nurses (6-10 years). Safety conditions most likely to be identified with sitter usage were delirium, elopement, and being a hospice patient. Sitters were more likely to identify falls risk, sitters 63% of time versus RN perception 30.9% (Chi Square=7.0, df=l, p=.008); dementia 59% vs 13% (Chi square=17.15, df=1, p=.001); and weakness 66.7% vs 18.2% (Chi square=16.54, df=1, p=.001). Sitters were more likely to have training in delirium 55% vs nurses, 34% (Chi sq=2.557, df=1 p=.11). Nurses identified that the use of sitters were very likely to prevent falls 29.8%, calm patients 25.2%, maintain lines 25.2%, prevent elopement 30.5% and redirect patients 29.7%. Nurses identified the following available safety strategies: alarms (67.2%), adjusting assignment (47.2%), music therapy (5.4%), use of restraints (<2%), pet therapy (<2%), and video monitoring (<1%). Implementation of safety programs must address availability of multiple strategies including: matching sitter competencies with patient populations served.

